# Immersive Virtual Reality and AI (Generative Pretrained Transformer) to Enhance Student Preparedness for Objective Structured Clinical Examinations: Mixed Methods Study

**DOI:** 10.2196/69428

**Published:** 2025-04-30

**Authors:** Shaniff Esmail, Brendan Concannon

**Affiliations:** 1 Department of Occupational Therapy University of Alberta Edmonton, AB Canada

**Keywords:** virtual reality, head-mounted display, immersive technology, artificial intelligence, generative pretrained transformer, occupational therapy, objective structured clinical examination, simulation, psychology, anxiety

## Abstract

**Background:**

Immersive virtual reality (VR) and artificial intelligence have been used to determine whether a simulated clinical exam setting can reduce anxiety in first-year occupational therapy students preparing for objective structured clinical examinations (OSCEs). Test anxiety is common among postsecondary students, leading to negative outcomes such as increased dropout risk, lower grades, and limited employment opportunities. Students unfamiliar with specific testing environments are particularly prone to anxiety. VR simulations of OSCEs may allow students to become familiar with the exam setting and reduce anxiety.

**Objective:**

This study aimed to assess the efficacy of a VR simulation depicting clinical settings to reduce student anxiety about a clinical exam while gathering perspectives on their first-year coursework experiences to better understand their learning environment.

**Methods:**

An experimental, nonrandomized controlled trial compared state anxiety, trait test anxiety, and OSCE grades in 2 groups of first-year occupational therapy students analyzed using independent *t* tests (2-tailed). Group 1 (NoVR) was not exposed to the VR simulation and acted as a control group for group 2 (YesVR), who were exposed to the VR simulation. The VR used artificial intelligence in the form of a generative pretrained transformer to generate responses from virtual patients as students interacted with them in natural language. Self-reported psychometric scales measured anxiety levels 3 days before the OSCE. YesVR students completed perceived preparation surveys at 2 time points—3 weeks and 3 days before the OSCE—analyzed using dependent *t* tests. Semistructured interviews and focus groups were conducted within 1 week after the OSCE. Student perspectives on their classes and VR experiences were summarized using interpretative thematic analysis.

**Results:**

In total, 60 students—32 (53%) in the NoVR group and 28 (47%) in the YesVR group—participated in the study, and the YesVR group showed a significant reduction in state anxiety (*t*_58_=3.96; *P*<.001; Cohen *d*=1.02). The mean difference was 11.96 units (95% CI 5.92-18.01). Trait test anxiety and OSCE scores remained static between groups. There was an increase in all perceived preparedness variables in the YesVR group. In total, 42% (25/60) of the participants took part in interviews and focus groups, providing major themes regarding factors that affect OSCE performance, including student experience and background, feedback and support, fear of unknown, self-consciousness, and knowledge of the exam environment.

**Conclusions:**

Intolerance of uncertainty may lead students to interpret ambiguous exam situations as overly precarious. Findings suggest that VR simulation was associated with reduced state anxiety, although results from this small, nonrandomized sample should be interpreted cautiously. Qualitative data indicated that VR helped students gain familiarity with clinical exam settings, potentially decreasing uncertainty-based anxiety. Future research with larger or randomized samples is needed to confirm these findings and explore advanced VR tools offering feedback to enhance learning.

## Introduction

### Background

This investigation was a mixed methods replication study expanding on a previous study by Concannon et al [1]. Their original work examined an immersive virtual reality (VR) simulation for occupational therapy (OT) students preparing for a clinical exam, observing a notable reduction in state anxiety 1 week before the objective structured clinical examination (OSCE). However, the aforementioned study did not collect qualitative feedback on VR’s benefits or examine how first-year coursework factors might influence performance, leaving open questions about which aspects of VR students found most useful and whether the results would generalize to new cohorts. Therefore, this investigation updated the technology using enhanced artificial intelligence (AI)–driven avatar responses and incorporated qualitative methods to better understand the student experience and confirm the consistency of the previous findings.

The purpose of this study was to replicate the results of the study by Concannon et al [1], expanding on the concept of student academic self-efficacy by measuring individual components of perceived preparedness, including student levels of confidence, awareness, knowledge, understanding, and skill level. This investigation used an improved VR simulation featuring the use of AI, which allowed the virtual patient to provide dynamic responses to student questions. This investigation also included qualitative components—semistructured interviews and focus groups were used to obtain student perspectives regarding their experiences with their first-year coursework and the VR simulation. These qualitative additions may offer context and student recommendations to better understand how immersive VR benefits first-year OT coursework.

### Campus Anxiety: Still a Prevalent Problem

Anxiety is an innate and typical response, preparing the body for an impending situation perceived to be dangerous [2]. It is a theoretical construct capable of manifesting in either general or specific situations, with *predisposition* (ie, trait anxiety) representing the frequency or intensity of the response and *transitory* (ie, state anxiety) representing an acute response to a specific situation and time [3]. Trait anxiety is associated with individual personality traits, impacting the level of state anxiety response within a specific context [3]. Test anxiety is also differentiated between *state test anxiety* [4]*,* showing transitory anxiety affects provoked by a specific evaluative situation, and *trait test anxiety* [5]*,* considered to be a general condition with varying ranges of intensity and contexts. Test anxiety has been defined by cognitive components, including negative thinking patterns (eg, *worry*) that may manifest negative thought outcomes and reduced attention, including the shifting of focus to irrelevant cues [6,7]. Worry thought processes include *catastrophizing,* where individuals have intrusive thoughts of failure or doubt in their capability [7].

There are also affective components, comprising *emotionality* aspects with physiological symptoms, including arousal and uneasiness (eg, stomach sensations, muscle tension, increased heart rate, headache, and sweating) [6,7]. These negative cognitive and emotional responses are strongly associated with reduced student performance on intellectual-based tasks [5]. There is a positive correlation between distracting contextual factors (ie, lack of dedicated study space and lack of access to reliable technology) and test anxiety [8]. Students who are unfamiliar with the test-taking environment for a particular exam are more likely to have symptoms of test anxiety as well [9]. It would be expected for students with previous experience in clinical practical exams, such as an OSCE, to show reduced anxiety due to improved familiarity. However, OSCEs have been identified as one of the most stressful exam types, with additional student exposure not necessarily lessening their anxiety about them in general [10,11].

The long-term effects of anxiety in postsecondary education include increased risk of dropout, reduced grades, reduced chance of employment, and the loss of billions in government dollars each year [12]. Test anxiety is associated with student burnout [13]. Campus anxiety is associated with depression and may be linked to increased student suicide rates [14-16]. Between 2008 and 2016, emergency department visits from students of US universities showed that anxiety complaints had increased by 260% [17]. In addition, test anxiety may predict the increase of student cheating behavior on exams [18]. One psychological model explaining why test anxiety occurs includes the intolerance of uncertainty model [19]. The intolerance of uncertainty model theorizes that people may inherently have an intolerance of uncertainty, resulting in ambiguous situations being perceived as threatening, leading to associated worry and anxiety [19,20]. While test anxiety is a normal phenomenon, it becomes problematic when students are unable to stop worrying or when the anxiety disrupts their practice [20].

### VR Versus Anxiety

A common method used to condition individuals against anxiety-inducing situations is in vivo exposure, in which an individual is exposed to situations or stimuli in real-world environments until distress is decreased [21]. VR extends this approach by reproducing real-world sights and sounds digitally, creating what is known as VR exposure therapy [21]. It is also known as *in virtuo* exposure [22]. VR may be preferable to traditional cognitive behavioral therapy as a means of exposure, functioning as an effective, affordable, and practical alternative to treatment avoidance [22].

A 3-step conceptual description of VR includes elements of user portrayal, immersion, and level of fidelity [23]. VR includes a broad range of computer-based programs, often used to render immersive and detailed 3D environments, allowing users to navigate within digital representations of the real world [24]. The goal of immersive VR is to replace the sensory flow of information from the real world with those created by a computer, promoting the illusion that the virtual world is real. The study and review by Concannon et al [1,25] provide more information about the immersive, interactive, imaginative, and fidelity abilities of immersive VR.

For cognitive adaptation (eg, improvement of memory, information processing, problem-solving, and logical sequencing), VR training has shown improvements in the brain’s frontal lobe and its cognitive characteristics, such as prompt recall of prospective memory tasks and precise performance of event- and time-based objectives [26]. VR learning may improve procedural memory because of changes in neural plasticity, improving working memory [27]. VR may improve a user’s cognitive and selective attention [28]. Enhancing problem-solving abilities and selective memory processes may improve social interactive elements such as those used in interview situations [29].

### VR in Health Science

A meta-analysis by Kyaw et al [30] found that health science students (ie, physicians, nurses, and students pursuing medical degrees) showed improvements in knowledge and skill outcomes when VR interventions were used (ie, 3D anatomical models, virtual patients, and surgical simulations) in comparison to traditional educational programs. Within OT education, VR simulations have gradually shifted toward more realistic, ecologically valid environments, especially for client intervention scenarios [31,32]. There is a need for mixed methods research that captures the nuanced perspectives and motivations of OT students, informing how VR can be most effectively integrated into their coursework.

### Aim of This Investigation

This study aimed to replicate the effectiveness of immersive VR in lowering state anxiety in OT students preparing for an OSCE. To uncover qualitative factors influencing student performance in coursework and VR simulations, interviews and focus groups were conducted. As an extension of academic self-efficacy, the students’ perceived preparedness factors for their OSCEs were tested to determine which had improved the most (ie, confidence, awareness, knowledge, understanding, and skill).

The objectives of this study were 2-fold: first, to quantitatively evaluate whether an AI-enhanced VR simulation was associated with reduced OSCE-related anxiety and, second, to gather student perceptions regarding VR’s usefulness, limitations, and potential enhancements. By integrating these perspectives, we aimed to refine the simulation’s design and determine how VR might be embedded into the broader OT curriculum to address both practical and emotional aspects of exam preparation.

### Research Questions

This investigation was designed to answer the following research questions: (1) does immersive VR simulation of a clinical practical exam (ie, OSCE) show a reduction in state anxiety in OT students when compared to a control group? (2) To better understand and improve learning systems, what are the student perceptions regarding VR simulation and OT coursework experiences? (3) Which variables of student perceived preparedness for their OSCE improved the most?

## Methods

### Experimental Design

This investigation was a mixed, cross-sectional, nonrandomized controlled trial involving 2 groups of participants, each comprising first-year OT students from the same class. This investigation attempted to replicate the difference in mean state anxiety between 2 groups of participants: students not exposed to a VR simulation (NoVR group), who acted as a control group for students who were exposed to VR (YesVR group). The University of Alberta Department of Occupational Therapy program had eased remote learning practice, which was established between the years of 2020 and 2022 in response to COVID-19 restrictions. First-year OT coursework remained consistent in terms of faculty infrastructure, course content, curricular sequencing, and instructors since the study by Concannon et al [1].

The mixed methods design was required for the two objectives: (1) to quantify any changes in state anxiety or exam performance following VR exposure and (2) to explore student perceptions of VR’s usefulness, limitations, and potential enhancements. This approach ensured that any observed quantitative shifts in anxiety could be contextualized with the qualitative insights essential for refining the simulation and shaping future curricular strategies.

This investigation assumed that student study time and strategies would not be significantly different between the 2 groups, omitting the need for students to provide written logs of their study strategies and time spent in preparation for the OSCE. This decision was implemented to minimize participant burden, forgoing the need for students to provide detailed study logs in addition to their course workload. The study by Concannon et al [1] used such logs and found that student time spent on study strategies for the OSCE was similar between groups, yet participants remarked that it was difficult to maintain the written logs in addition to regular coursework.

### Recruitment

At the start of the term, a speaker who was neutral to the investigation’s outcome made in-class announcements providing details of the investigation. The speaker was not an instructor from the faculty; this was to minimize the compulsory pressure on students to participate. The announcement was made during a lecture to an OT class of 125 first-year students. All OT students were invited and eligible to participate. Students were informed of the class email announcements and invitations to web-based surveys, which would be made available for them to accept and participate. The speaker concluded the announcement with a brief demonstration of the VR hardware that was available, including basics on how to use it and the risks typically associated with VR use.

### Ethical Considerations

This study was reviewed and approved by the University of Alberta’s Research Ethics Office (Pro00107738) in accordance with the Tri-Council Policy Statement: Ethical Conduct for Research Involving Humans. All participants received an information letter outlining the study purpose, procedures, and potential risks, and they provided informed consent via signed or electronic forms. To protect privacy, data were deidentified at collection and stored in encrypted files accessible only to designated research assistants; any identifying details were removed from transcripts or replaced with aliases to ensure anonymity in all reports and publications. No compensation was offered, and use of the VR simulation was made available regardless of participation. This manuscript does not include images that could identify individuals. Any similarity to actual persons is purely coincidental. Although demographic data on sex are often collected in studies of this nature, the reviewing ethics board restricted these measures to protect individuals’ anonymity given the program’s enrollment composition (approximately 90% female, 8% male, and 1% identifying otherwise). As a result, sex-based data were not gathered.

### Experimental Process

**Figure 1:**
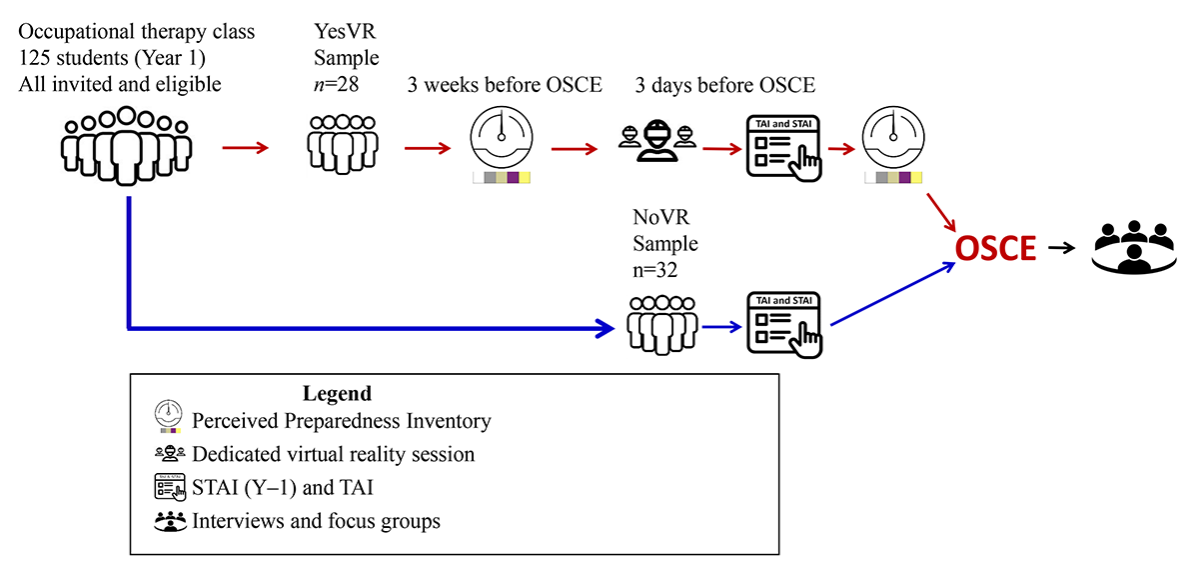
Experimental process of this investigation. NoVR: participants not exposed to the virtual reality simulation; OSCE: objective structured clinical examination; STAI: State-Trait Anxiety Inventory; TAI: Test Anxiety Inventory; YesVR: participants exposed to the virtual reality simulation.

In this investigation, while the VR simulation was available as a self-serve tool, it was important to have a consistent measure taken at the peak anxiety time point. This investigation used class email announcements to offer a consolidated VR session appointment. Those who accepted the offer were designated as YesVR participants and administered a web-based Perceived Preparedness Inventory survey 3 weeks before the OSCE. The appointed YesVR students were given their scheduled VR sessions 3 days before the OSCE, followed by a Perceived Preparedness Inventory survey again. There were separate rooms, each equipped with VR headsets, allowing multiple participants to privately complete the simulation simultaneously. If one headset had a technical difficulty, participants could be assigned to another room to keep the process expeditious. After their VR appointments, a class email announcement was sent out to invite all students to complete web-based State-Trait Anxiety Inventory (STAI) and Test Anxiety Inventory (TAI) surveys measuring student state anxiety and trait test anxiety levels, respectively. These surveys asked students whether they had completed the VR simulation. Students who reported not having used the VR simulation yet chose to complete the STAI were designated as the NoVR group. The timing of the web-based STAI, TAI, and Perceived Preparedness Inventory surveys was set to take place after the VR sessions and class hours so that students could complete them outside the presence of instructors or researchers. Focus groups and interviews were scheduled within a 1-week time frame after the students completed their OSCEs via class email announcements and web-based sign-up sheets. Figure 1 provides a summary of this investigation’s experimental process.

### Primary Outcome Measures

#### STAI Forms

The STAI is divided into 2 forms: the Y-1 (S-Anxiety) scale, which measures a participant’s level of anxiety at a specific moment in time, and the Y-2 (T-Anxiety) scale, which measures a participant’s predisposition and long-standing level of anxiety. Each form comprises 20 items, with final scores ranging from 20 to 80 and higher scores representing greater levels of anxiety [3]. The STAI S-Anxiety scale was developed to measure college students’ levels of state anxiety, showing a reliability stability of *r*<0.62. Reliability coefficients for this instrument are expected to be low to moderate due to its capability to show differences in participant anxiety levels between each testing-retesting situation [3]. The STAI S-Anxiety scale normative Cronbach α coefficients for college students have been reported to be 0.91 and 0.93 for male and female individuals, respectively [3]. For validity, the STAI S-Anxiety scale was compared to the Institute of Personality and Ability Testing anxiety scale and Taylor Manifest Anxiety Scale, showing validity correlation coefficients of *r*=0.75 and *r*=0.80, respectively [3]. Overall, the STAI shows consistency in measuring defined components of anxiety, including apprehension, tension, nervousness, and worry [3]. For this investigation, the STAI S-Anxiety scale was administered as a web-based survey to both the YesVR and NoVR participants at the same time point, 3 days before the OSCE.

#### TAI Forms

The TAI is a subjective psychometric scale measuring individual differences in anxiety about testing situations as a personality trait. The instrument consists of 20 items, with final scores ranging from 20 to 80 and higher scores being associated with stronger symptoms of test anxiety [33]. The TAI scale has shown a reliability stability of *r*=0.80 for periods varying between 2 weeks and 6 months [33]. The TAI showed normative Cronbach α coefficients for college students of 0.92 for both male and female individuals [33]. For validity, the TAI was compared to the Test Anxiety Scale and Worry-Emotionality Questionnaire, showing validity coefficients of *r*=0.83 and *r*=0.85, respectively [33]. For this investigation, the TAI was administered as a web-based survey to both the YesVR and NoVR participants at the same time point, 3 days before the OSCE.

#### Interview and Focus Group Setup

After the OSCE, all students received an email inviting them to sign up for interviews and focus groups, which remained open for 1 week. Participation was voluntary and not restricted to those who had used the VR simulation. To uphold ethical guidelines and prevent any sense of obligation, all students were encouraged to share their experiences regardless of VR participation. The interviews and focus groups lasted approximately 45 to 60 minutes. Copies of the interview and focus group questionnaires are available in Multimedia Appendices 1 and 2, respectively. Interview and focus group data were summarized using an interpretative thematic analysis based on an approach developed by Burnard [34], the interview process guide by Kvale [35], and the general approach by Maykut and Morehouse [36].

Participant responses were grouped into various categories as recurring trends emerged. Analysis of field notes and transcripts was processed across 10 stages partitioned into 3 phases: phase 1, consisting of 4 stages in which the data were filtered and categorized; phase 2, consisting of 3 stages in which data were condensed, organized, and integrated; and phase 3, in which understanding and meaning were derived from the data. This structured, 10-step and open coding process primarily aligns with the approach by Burnard [34], whereas the constant comparison method aligns with the approach by Maykut and Morehouse [36] and the emphasis on interpretation and participant perspectives by Kvale [35]. Table 1 provides more information on the interview and focus group analysis process.

In keeping with recommendations by Maykut and Morehouse [36], the research assistants maintained reflexive journals to document their assumptions and any shifts in interpretation. In total, 2 researchers independently reviewed the transcripts, reconciling coding differences through iterative discussion, thereby supporting confirmability [34]. Dependability was strengthened by maintaining an audit trail of analytic decisions, as outlined by Maykut and Morehouse [36].

**Table 1:** Interview and focus group analysis process.

Phase and stage			Process
**Phase 1: filtering and categorizing**			
	1	Notes are written immediately after the interview. Memos are written about ways of categorizing data.	
	2	Each transcription is read to gain a better understanding of some of the larger themes. Quick notes are made (eg, terms and definitions).	
	3	Each transcript is read again, and detailed notes on general themes and major categories are recorded in table format.	
	4	Open coding [34]: categories are created as needed to describe what is in the text. Transcriptions are read again, and as many headings and themes as possible are recorded. Fillers are deleted.	
**Phase 2: condensing, organizing, and integrating**			
	5	Constant comparison method [36]: data segments are continuously compared to emerging categories. Data meanings are compared to each new unit and existing units before grouping. Data are condensed, organized, and integrated. Headings and themes are narrowed down, and repetitive ones are removed.	
	6	Headings and themes are grouped under higher-order headings.	
	7	Transcriptions are reviewed again using the categories and higher-order headings.	
**Phase 3: understanding and meaning**			
	8	Discovery pages are created with categories listed and themes grouped under them. Short narrations briefly describing the primary points are created. Comparisons are made between interviews.	
	9	Narratives are shared with participants or within the research team to refine the data.	
	10	Narratives are linked with what is found in the literature and how it relates to the research questions.	

### Secondary Outcome Measures

#### Perceived Preparedness Inventory

The aim of this investigation was to analyze individual components of student perceived preparedness, including self-confidence, awareness, knowledge, understanding, and skill level in accordance with OT learning objectives. The Perceived Preparedness Inventory survey was used to assess these specific components, allowing for the observation of which variables improved the most throughout the term. This web-based survey was adapted from the physiotherapy study by Weeks and Horan [37] and the nursing study by Massey et al [11] and used a 5-item, 5-point Likert scale to determine the variables that showed the strongest improvements in student perceived preparedness within their OT coursework. The survey’s wording was intended to probe students’ perceived preparedness variables for OSCEs in a general sense and did not address the impact of immersive VR. A copy of this survey is available in Multimedia Appendix 3.

#### OSCE Scores

OSCE score comparisons between the NoVR and YesVR groups were conducted to determine the effects of the VR simulation on student performance. Specifically, comparisons were made between the 2 groups’ history taking and cognitive assessment OSCE scores, as well as their total course grades. During the OSCE, each student was required to complete 2 stations followed by a written component. The first station required students to interact with a standardized patient (actor) who was afflicted with a random physical ailment, making up the OSCE’s history taking and physical check component. This random physical complaint (scenario) would be an acute injury in a random peripheral anatomical location. The second station required students to interact with a different standardized patient representing a client with a mild cognitive disability. After these 2 stations were completed, the students would complete a written component requiring them to apply their clinical findings, demonstrate critical thinking, and clarify their clinical reasoning related to the scenario.

### Simulation Design

#### Overview

The VR simulation in this investigation included the following components:

- A virtual environment depicting a health sciences clinic rendered using the Unity game engine software (Unity Technologies). The environment allowed the user to select from 1 of 2 modules: History Taking or the Saint Louis University Mental Status (SLUMS) exam cognitive assessment [38].
- 3 virtual avatars—the first appeared in the History Taking module as a virtual standardized patient who responded to a user’s questions, the second appeared in the cognitive assessment module as a virtual standardized patient who responded to a user question from the SLUMS cognitive assessment, and the third was a virtual exam evaluator who observed the user and wrote notes into a clipboard during each module.
- Speech recognition and response software using Azure Cognitive Services (Microsoft Corp). The process pipeline includes user speech to text, open AI for language processing and generation of the virtual avatar’s text response, and the avatar’s text converted from text to speech.
- Meta Quest 2 headsets that ran the VR software, uploaded using SideQuest (Khronos Group). These headsets were portable and free of cables. The headset could detect a user’s hand gestures without the use of controllers. The headset was also equipped with a microphone to detect user speech for communicating with the virtual avatars.

#### Virtual Health Sciences Clinic

This investigation rebuilt the VR simulation based on the system mentioned in the study by Concannon et al [1] using the same interdisciplinary team members’ expertise in computer science, physical therapy, communication and science disorders, rehabilitation medicine, and OT. Student and researcher feedback from the aforementioned study was implemented to further improve the system used in this investigation. The virtual clinic in this investigation had the same clinical office layout as that used in the previous study, yet this simulation had graphical, functional, and quality-of-life improvements. The virtual clinic’s tools, tables, and plinth layout were arranged to closely match those used in the actual OSCE room. This study’s virtual clinic had a main door with a menu. This menu allowed users to select the virtual module they wanted to attempt: History Taking or the SLUMS exam. The user could do this by using hand gestures to push buttons on the door, which the headset could detect. Upon selecting an option, the door would open to a hall where the user could walk to the examination room to meet the standardized patient.

**Figure 2:**
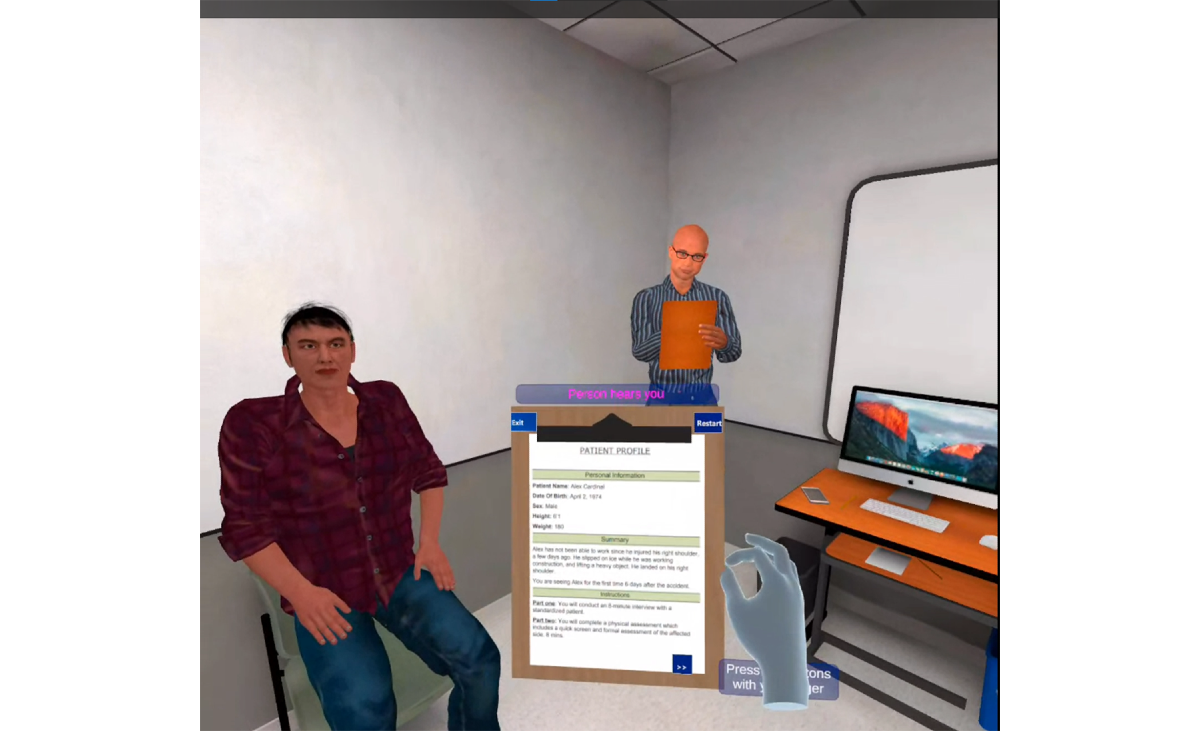
Screenshot of the virtual History Taking module.

Depending on the module, the virtual clinic had a different clipboard for the user to interact with and read from. The History Taking clipboard contained preliminary notes about the virtual patient, similar to what the student would receive before interacting with their real-world OSCE standardized patient. The virtual patient in this module had a random physical complaint (scenario), such as an acute injury in a random peripheral anatomical location. Once the user entered the virtual room, a buzzer sounded to signal the start of the virtual OSCE, and a miniature timer on a desk began counting down from 8 minutes. The user could ask the virtual patient questions in natural language, with the user’s voice being detected by the headset’s microphone to convert the question from speech to text. The virtual patient would respond to health questions, including those about physical, social, psychological, spiritual, emotional, workplace, and daily living health domains. Figure 2 provides a sample screenshot of the History Taking module.

For the SLUMS module, the clipboard contained the questions and scoring rubric from the SLUMS cognitive assessment. A copy of the SLUMS cognitive assessment and scoring rubric has been included in Multimedia Appendix 4. The virtual patient in the SLUMS module would attempt to solve each test question asked by the student, with a random 25% chance of error. For example, one question required the student to request the virtual patient to draw the hands of a clock inside a circle, showing a time of 10:50. The virtual patient would answer appropriately, conducting the motion of drawing the clock on a piece of paper to display an illustration of a clock with hour and minute hands indicating a time of 10 minutes to 11. There was a 25% random chance that the virtual patient would make a mistake while attempting this task, drawing the clock with the hour and minute hands in switched positions or the incorrect time altogether. The SLUMS module’s miniature timer began counting down from 15 minutes once the module started as the user entered the virtual room.

Real-world OSCEs are typically station oriented, attempting to replicate clinical scenarios with as much detail as possible. They usually feature standardized patients who represent clinical patients in health-related situations. A typical OSCE will grade a student’s performance across an array of evaluation categories, including (the percentages represent mean scoring components) [39] history taking (30%-40%), physical examination (20%-40%), patient education (0%-10%), physician-patient interaction (20%-40%), and note practice (5%).

#### Speech Recognition Software

The virtual standardized patients’ text responses were generated using a generative pretrained transformer (GPT; GPT-3.5 [OpenAI]), which was fine-tuned (ie, trained) on recorded live student and patient actor interactions. GPT models use a neural network that has undergone pretraining using a substantial literature database. When a GPT creates text, it uses learned information to choose the most likely subsequent word based on the previous words in a sequence, aligning with the word probability distribution learned from its training data. This procedure is reiterated for each following word in the sequence, leading to the formation of a naturally flowing sentence. For this investigation, the avatar training was conducted using a transfer learning technique on the base model using real-world student and standardized patient text files that were collected from transcribed recordings. The simulation’s GPT-3.5–based model was deployed on the Azure Machine Learning Cloud service (Microsoft Corp) for real-time inference. Virtual avatar actions (behaviors) were generated in text form by GPT-3.5 and then linked to appropriate animations, allowing them to respond to user requests (eg, if a user asked the virtual patient the following— “Can you raise both arms up into the air?”—the virtual avatar would respond accordingly). GPT-3.5 selected appropriate avatar action and verbal responses deployed through the Azure Machine Learning Cloud service using custom training and deployment scripts. In other words, GPT-3.5 acted as the actual *language process and avatar action–deciding* engine. Azure Machine Learning Cloud functioned as a bridge, relaying information between the VR headset and the GPT. The virtual patient could provide basic responses to convergent questions. For instance, if a user said the following—“Can you tell me more?”—the virtual patient could respond with related information based on the previous question or simply reply, “That’s all I know.”

### Statistical Analysis

Statistical significance was evaluated at α=.05, and a 2-sided *P* value of ≤.05 was considered to be statistically significant. Comparisons between the NoVR and YesVR groups were conducted using independent *t* tests (2-tailed), which compared STAI, TAI, and OSCE scores between the groups. Repeated *t* tests were used to compare the YesVR group’s perceived preparedness results before and after the VR sessions. The Cohen *d* effect size was checked for each *t* test between the groups.

## Results

### Overview

A total of 60 students participated in the quantitative portion of this study: 32 (53%) in the NoVR group and 28 (47%) in the YesVR group. A total of 25 students participated in the qualitative portion of this study: 16 (64%) in the interviews and 9 (36%) in the focus groups. Most YesVR participants (26/28, 93%) used the VR simulation for both modules, which typically meant 8 minutes of the History Taking module and an additional 15 minutes with the SLUMS module, in addition to some participants retrying one or both of the modules. The mean VR simulation time spent by the YesVR group was 24.11 (SD 8.00) minutes per participant. Table 2 provides the participant data. The main statistical analysis results are provided in Table 3. Perceived‑preparation results for the YesVR group are presented in Table 4.

**Table 2:** Participant data.

Group		Participants, n (%)
**Quantitative portion (n=60)^a^**		
	NoVR^b^ participants^c^	32 (53)
	YesVR^d^ participants^e^	28 (47)
**Qualitative portion (n=25)**		
	Interview participants	16 (64)
	Focus group participants	9 (36)

^a^Mean age 24.53 (SD 2.64) years.

^b^NoVR: participants not exposed to the virtual reality simulation.

^c^Mean age 24.52 (SD 2.42) years.

^d^YesVR: participants exposed to the virtual reality simulation.

^e^Mean age 24.54 (SD 2.93) years.

**Table 3:** Statistical analysis results.

Test	NoVR^a^, mean (SD)	YesVR^b^, mean (SD)	*t* test (*df*)	*P* value	Cohen *d*
STAI^c^ (form Y-1) score	55.00 (10.18)	43.04 (13.19)	3.96 (58)	<.001	1.02
TAI^d^ score	41.81 (14.36)	43.50 (13.13)	−0.47 (58)	.64	0.12
OSCE^e^ score: history taking	86.38 (6.53)	87.25 (7.64)	0.48 (58)	.63	0.12
OSCE^e^ score: cognitive assessment	88.47 (8.04)	90.07 (5.88)	0.87 (58)	.39	0.23
OSCE^e^ score: course total	90.03 (4.26)	90.25 (4.92)	0.19 (58)	.85	0.05

^a^NoVR: participants not exposed to the virtual reality simulation.

^b^YesVR: participants exposed to the virtual reality simulation.

^c^STAI: State-Trait Anxiety Inventory. Measure taken 3 days before the Objective structured clinical examination.

^d^TAI: Test Anxiety Inventory. Measure taken 3 days before the objective structured clinical examination.

^e^OSCE: objective structured clinical examination.

**Table 4:** Perceived preparation analysis results.

Preparedness domain	Pre-VR^a^, mean (SD)	Post-VR^b^, mean (SD)	*t* test (*df*)	*P* value	Cohen *d*
Perceived preparedness: confidence	2.71 (0.94)	3.46 (1.00)	2.68 (27)^c^	.01	0.77
Perceived preparedness: awareness	3.18 (1.16)	3.89 (0.86)	2.68 (27)^c^	.01	0.70
Perceived preparedness: knowledge	2.82 (0.82)	3.61 (1.03)	3.16 (27)^c^	.004	0.85
Perceived preparedness: understanding	3.32 (0.90)	3.93 (0.98)	2.62 (27)^c^	.01	0.65
Perceived preparedness: skill	3.00 (0.98)	4.04 (0.96)	4.16 (27)^c^	<.001	1.07

^a^PreVR: student scores 3 weeks before the objective structured clinical examination, before their virtual reality training session.

^b^PostVR: student scores 3 days before the objective structured clinical examination, after their virtual reality training session.

^c^Participants who completed the perceived preparedness surveys were in the YesVR group only; thus, df were changed accordingly.

### Primary Outcome Measures

#### State Anxiety Scores

There was a significant difference in state anxiety scores between the groups, with NoVR showing higher anxiety scores (mean 55.00, SD 10.18) than YesVR (mean 43.04, SD 13.19; t_58_=3.96; *P*<.001; Cohen *d*=1.02). The mean difference was 11.96 units (95% CI 5.92-18.01).

#### Test Anxiety Scores

There was no significant difference in test anxiety scores between the groups, with NoVR showing similar anxiety scores (mean 41.81, SD 14.36) to those of YesVR (mean 43.50, SD 13.13; t_58_=−0.47; *P*=.64; Cohen *d*=0.12). The mean difference was −1.69 units (95% CI −8.84 to 5.46).

#### Student Perspectives and Experience

#### Overview

A total of 42% (25/60) of the students participated in the interviews and focus groups. Each of the 8 interview sessions had 2 participants interviewed separately. The first focus group session had 5 participants, whereas the second focus group session had 4 participants. Participants remarked that the actual exam was not as difficult or challenging as they thought it would be. Participants also remarked that time management was the most stressful part of the OSCE. VR was cited as being useful in helping with student orientation regarding the exam procedure while allowing students to fail and work through difficulties in a low-stakes environment. Regarding areas of improvement, students stated that they wanted improved feedback from the VR simulation and the ability to practice components of the OSCE physical check. Table 5 provides a summary of the interview and focus group themes.

**Table 5:** Interview and focus group theme analysis summary.

Themes		Subthemes
**Major themes**		
	Experience and background	Previous clinical exam experience Personality traits (talkative vs reserved)
	Need for feedback and support	Instructor and peer feedback Laboratory size (number of students) challenges
	Fear of the unknown or unexpected	Anxiety about uncertain scenarios Unexpected events during the OSCE^a^
	Self-consciousness and insecurities	Comparing oneself to peers Heightened anxiety when observed and fear of failure
	Knowledge of the exam environment	Importance of knowing the exam room ahead of time Useful for the VR^b^ exam setting to match the exam room layout
	Practice should mimic the actual exam	Formal note-taking aligned with the OSCE format Graded mock OSCE sessions to reduce anxiety
	Student grade mindset	Viewing the OSCE as “just another test” vs a major assessment Impact of learning vs performance focus
	Time constraint	Rushed feeling during the written component Differences between class practice time and OSCE format
	Peer influences	Social atmosphere in group practice as exams drew near Peer influence on VR uptake (encouraging or discouraging)
**Minor themes**		
	Framing	Impact of how the faculty present the OSCE Mentally reframing the exam (game-like vs high stakes)
	Pace of practice laboratories	Laboratories perceived as too fast Inadequate time to practice new skills
	Inconsistencies	Contradictory instructions in coursework VR avatar responses that do not align with questions
	OSCE not realistic	Delays in standardized patient responses Exam setting differs from real-world clinical practice

^a^OSCE: objective structured clinical examination.

^b^VR: virtual reality.

#### Major Themes

### Experience and Background

Most students indicated that previous experience and background were factors influencing OSCE performance. Students said that having experience with clinical exams would lessen exam stress. As one student said, “I think the reason that the OSCE is quite stressful is because many of us have not done a practical exam like this.” Other students said that their comfort with the OSCE was influenced by their previous education, work experience, or personality. For instance, students said that “having previous knowledge or possessing a degree in anatomy helped [us] prepare for the physical assessment more than students without.” There were students who were more confident with the history taking because of “the previous work experiences and interactions that [they] have had.” Regarding personality, one student believed that she did well in the history taking because she was naturally “a talkative, sociable person,” whereas another student felt that she would struggle because she was “not hugely a people person.” A student remarked that people with gaming experience may help them appreciate the VR simulation.

### Need for Feedback and Support

Another consistent theme was the mention that any practice sessions in which students received feedback were especially helpful, such as the practice history-taking sessions with the senior students and the mock OSCEs in which instructors graded students using the marking rubric. In class, some students felt that the class sizes made it more difficult to receive feedback:

...to get feedback was not always easy because there is only one instructor to how many students?

Due to the lack of immediate feedback, a few students expressed that it may be beneficial to be paired with a laboratory partner who has more experience and knowledge on physical assessments. This way, novices could receive quality feedback when needed as opposed to practicing with someone else who is not as familiar with the content. Overall, students said that having access to timely feedback and support played a main role in their sense of preparedness. Regarding the VR simulation, a focus group unanimously agreed (with nodding and affirmative gestures) that the VR modules provided feedback in an indirect manner:

VR made us be more aware to ask simpler questions in the OSCE, because the VR would glitch-up if we talked too much.

When probed about this further, students said that the glitches would often result in the virtual patient saying an off-topic remark, such as “I like to play ice-hockey!” One student said that she found the VR to be stress relieving because the glitches were funny and made her laugh. Most VR students recommended that the simulation be upgraded to provide immediate feedback.

### Fear of the Unknown or Unexpected

Another theme that emerged involved students mentioning feeling anxious and nervous about the OSCE because they did not know what to expect. One of the main sources of stress was not knowing which scenario they were going to be assigned ahead of time. As one student said, “I dreaded the scenario selection because you didn’t know what you were going to get.” Other students felt stressed because they were unfamiliar with the clinical exam process, such as not knowing the test environment or procedure. Notably, several students had unexpected events happen during their OSCEs, which contributed to their sense of stress. For example, one student’s client was an instructor she knew, whereas another student said that “my client kept looking at the evaluator.”

### Self-Consciousness and Insecurities

Another theme that emerged involved students’ feelings of self-consciousness, insecurity, and stress, especially when they compared themselves to their classmates. These students admitted to forming comparisons without regard to study progress, knowledge, and competency. Some students felt intimidated when they saw that their classmates had started preparing for the OSCE before them:

...it made me anxious to know how early the people were starting

to study

Others felt incompetent and doubted their ability to pass the test. Notably, for one participant, the stress from comparing herself to her peers was even greater than the actual OSCE. As a subtheme, other participants said that their self-consciousness manifested whenever they felt fear of being observed and the fear of failure. A few students feared being watched because they associated those situations with judgment (ie, being evaluated). These students worried that they would be deemed incompetent. Some students had fears of failing the OSCE and what others would think of them. One student described the following:

I didn’t want to fail because I didn’t want to look incompetent to my peers, more so than me actually caring for my own sake.

Another student made a similar remark:

...no one wanted to be known as the person who failed it.

### Knowledge of the Exam Environment

Another consistent theme was the importance of having knowledge of the OSCE room before the exam. These students believed that it would be helpful to be shown the exam room ahead of time, whether through a tour or through practice sessions in it. Specifically one student stated they would be “more thrown off by the foreign environment.” Another student made a similar remark, stating that the move from a classroom (practice) to a clinical (exam) setting was intimidating. As one student described, the differences between the room where she conducted her practice interview with second-year students and the actual exam room initially “threw me off.” In total, 8% (2/25) of the students said that they had difficulty operating the plinths or locating tools in the actual OSCE room. One student said that it would be useful to see the environment ahead of time so that she could visualize the room and rehearse. Several students recommended that simulated exam rooms mimic the actual test environment as closely as possible. Regarding the VR simulation, many students said that VR helped them visualize what the actual OSCE would look like. One student remarked the following:

I found it helpful. [It was] my first VR experience. I got to see what the layout of the room would be like.

### Practice Should Mimic the Actual Exam

Another major theme that emerged related to practicing for the OSCEs. The students recommended that their practice should closely mimic the actual exam as much as possible. With regard to the standardized patients, some students believed that practicing with classmates was less useful because “there is an expectation there where if I give [a classmate] a little prompt, they should be doing what I asked. Whereas if you do it with a patient, they don’t know what you are talking about unless you give them full instructions.” One student remarked that there were few male students in the course, which resulted in increased feelings of nervousness during the OSCE because her practice was only on female students. Several students remarked that they did not have enough practice with formal documentation of clinical notes in class. They further mentioned that note-taking was “different” between in-class and exam sessions:

In the OSCE, [we] have 30 minutes for two notes and three questions.

Some students remarked that practice notes, done in laboratories, did not realistically fulfill the expectations of the OSCE. Students found practice sessions useful when instructors would grade them based on the rubric. One of the students remarked that, after she did a practice OSCE in front of the class and was marked on it, she felt more relieved because “you realize there’s room to make mistakes.” Regarding the VR simulation, one student pointed out that *VR gave us different ways to ask questions.* Another student remarked the following:

I think it would be helpful to reiterate the fact that VR is a different way to practice for the OSCE.

### Student Grade Mindset

Students’ mindsets regarding the OSCE impacted their perceived anxiety and response. Some students mentally *equalized the weight* of the OSCE by saying the following:

I think anything [worth] over 10% as the same in my world. You have to treat it just like any other test.

These thought processes were admitted by students to potentially reduce their anxiety about the OSCE. In contrast, others viewed it more as a learning opportunity:

I’ve viewed it just as a learning experience. I don’t think I really look at [the OSCE] as the 50% or maybe the 30% for human systems or whatever.

Some students agreed that viewing the OSCE as only a learning experience felt less distressing to them, saying the following:

...if I did something poor enough to actually fail, then I hope I would get corrected and learn how to do it right.

In contrast, one student said that her peers were “...all super high achieving, wanting to be the best kind of student,” which led to her feeling scared about the possibility of failing. Regarding the VR simulation, one student remarked the following:

I would try any practice method presented to me. I think VR helped me chill out a little. I think in the future, in general, I’ll try to remember this.

Although the students’ mindsets impacted their perceived anxiety, when they focused on the grades attached to the OSCE, they admitted to having potentially greater anxiety levels than those who viewed the OSCE as a learning activity only. Students admitted that the VR simulation may have helped them reduce their anxiety as it functioned as a stand-alone learning activity in this manner.

### Time Constraint

A common theme reported by students was admitting to being under pressure to complete the OSCE on time, which felt rushed. In particular, many students found the written component to be the most fast-paced. For instance, one participant said the following:

I ran of time, when I was answering the questions, so I knew my answers sucked...[I] had two minutes to answer all three questions so I just wrote whatever came into my mind.

A significant number of students felt that the time allotted was not enough given what they were expected to complete. One student asked the following:

...how can we write two notes and answer the questions in twenty minutes?

The students also mentioned that the time on the OSCE was not similar to that in the practice sessions they had received in class. Notably, a participant expressed the following:

...in class, we have twenty minutes to write just a SOAP note and you are still feeling rushed. Whereas in the OSCE, you have about fifteen-ish minutes to do each.

While one student mentioned running out of time during the physical check, most remarks on time constraints were about the written test.

### Peer Influences

A theme emerged regarding the perceived change in the social atmosphere as the appointed time of the OSCE drew near, with statements such as the following:

...the vibe of the class nearing the OSCE made it more stressful.

One student said the following:

...being in the group setting while everyone was practicing, you got that very nervous environment going on and everyone’s asking questions about how you do this and that. I thought that this was spreading fear in our class.

One student said that peer pressure was more anxiety inducing than the upcoming OSCE itself. She remarked the following:

...it wasn’t even the OSCE [causing anxiety], because as long as you are practicing and you prepared yourself, I felt it was okay. But it was the pressure from everyone else to be like we’ve got to do this, this, and this.

Another student said that peer perception added to anxiety, leading to some students avoiding their peers altogether. Despite mentions of negative peer influences, students reported that their classmates were also helpful in offering assurances and knowledge. Students were influenced by their peers to attempt or refuse the VR simulation based on how effective they believed it was. For example, one student said the following:

Friends that did VR were suggesting people to not take VR. After hearing [about] other people’s experiences, some decided to back out.

#### Minor Themes

### Framing

An additional theme emerged indicating how the OSCE could have been framed differently by the faculty. Students suggested that the instructors could have introduced the OSCE in a less anxiety-provoking way. Students mentioned that bringing up the OSCE on the first day of class in the academic year for first-year students caused them stress. One student mentioned the following during a focus group (acting in panic to represent their mood at the time): “What is this?” and “How is this half of my mark?” Students suggested that the faculty could make the OSCE less intimidating by changing the wording to make exams sound less important (eg, calling a midterm a quiz) and avoiding the use of acronyms. In total, 8% (2/25) of the students said that they mentally reframed the OSCE, lessening their anxiety over it. Regarding the VR simulation, one student pointed out the following:

...for some people, seeing the OSCE as a video-game or something fun to engage with, rather than all this pressure...it could create a shift in some people.

### Pace of Practice Laboratories

Students also felt that the practice laboratories were too fast, which failed to allow students adequate time to learn and practice the content:

The speed of the content [coursework] was a problem.

Students in both interviews and focus groups mentioned that the material in the laboratory was taught too fast, which made it harder for them to learn and remember. These students said that they did not feel as if they had adequate time for both partners to practice a task, leaving some students confused after the laboratories. One student remarked the following:

...labs in general always felt kind of rushed, so when we practiced, we’d only get fifteen minutes and then, okay, we are starting [to learn new content] again.

### Inconsistencies

Other concerns raised by students that added to their stress and anxiety were being given inconsistent information. According to these students, there were inconsistencies in both course delivery and exam marking. While they acknowledged that such inconsistencies were eventually cleared up, one felt *stressed and confused by the ordeal.* One student added the following:

...having to re-learn a ROM or MMT movement, due to being told incorrect information, was highly inefficient.

Regarding the VR simulation, inconsistencies involving the virtual patient’s responses were also reported:

I asked the VR patient if he was a father and he responded by saying “not really.” And then as time went on, I figured out he had two children, so yeah, he was a father.

### OSCE Not Realistic

Another theme that emerged was that students felt that, while the actual OSCE was designed to represent a real-world clinical exam, it did not accurately reflect everyday practice. During the physical check on a standardized patient, one student thought that looking for a single impaired movement was unnatural because rarely would an injury only affect 1 movement in real life. Students also noted a “lag time between asking a question and waiting for the patient to reply.” Another 8% (2/25) of the students commented on the time limit for the interview:

[the] timeframe threw me off a bit—it seemed so inorganic. Like, it won’t be like that in real life, probably.

Regarding the VR simulation, one user said the following:

I think the simulation did not accurately reflect what it was going to be in the [actual] OSCE. Even if it did, every OSCE is different, so it may not help so much for the future.

#### Secondary Outcome Measures

##### Perceived Preparedness Inventory

**Figure 3:**
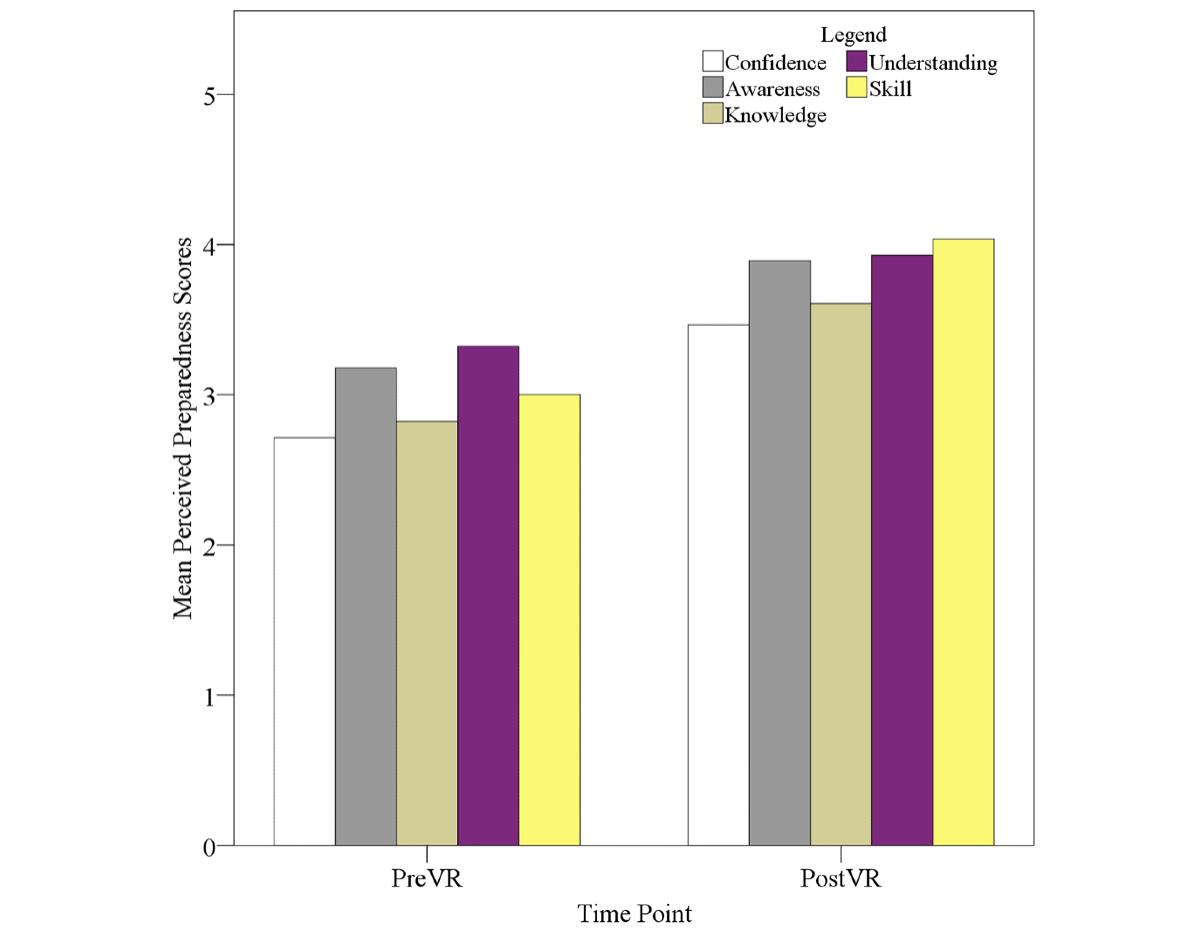
Student perceived preparedness scores. PreVR: measure taken 3 weeks before virtual reality simulation; PostVR: measure taken after virtual reality simulation.

Figure 3 shows student Perceived Preparedness Inventory scores for student confidence, awareness, knowledge, understanding, and skill levels. There was a significant increase in all variables from before to after VR. It should be noted that the presentation of scores in this report for perceived preparedness was arranged with higher values representing greater mean levels of student-reported ability, unlike the traditional scoring of this instrument, which represents lower scores as being better. There was a significant difference in perceived confidence between mean scores before VR, which showed the lowest values (mean 2.71, SD 0.94) out of all variables, and mean scores after VR (mean 3.46, SD 1.00; t_27_=2.68; *P*=.01; Cohen *d*=0.77), which were also the lowest of all variables at that time point. The mean difference was 0.75 units (95% CI 0.18-1.32). Perceived awareness showed a significant difference from before VR (mean 3.18, SD 1.16) to after VR (mean 3.89, SD 0.86; t_27_=2.68; *P*=.01; Cohen *d*=0.70), with the mean difference being 0.71 units (95% CI 0.17-1.26). Perceived knowledge scores showed a significant mean increase from before VR (mean 2.82, SD 0.82) to after VR (mean 3.61, SD 1.03; t_27_=3.16; *P*=.004; Cohen *d*=0.85), with the mean difference being 0.79 units (95% CI 0.28-1.30). Of all the variables, understanding showed the highest mean scores before VR (mean 3.32, SD 0.90) and the second-highest mean scores after VR (mean 3.93, SD 98; t_27_=2.62; *P*=.01; Cohen *d*=0.65). The mean difference was 0.61 units (95% CI 0.13-1.08). Finally, student-perceived skill showed the greatest significant increase from before VR (mean 3.00, SD 0.98) to after VR (mean 4.04, SD 0.96; t_27_=4.16; *P*<.001; Cohen *d*=1.07) and also showed the highest mean scores at that time point. The mean difference was 1.04 units (95% CI 0.52-1.55).

##### OSCE Scores

There was no significant difference in mean OSCE scores between the NoVR group (history taking: mean 86.38, SD 6.53; cognitive assessment: mean 88.47, SD 8.04; course total: mean 90.03, SD 4.26) and the YesVR group (history taking: mean 87.25, SD 7.64; cognitive assessment: mean 90.07, SD 5.88; course total: mean 90.25, SD 4.92; history taking: t_58_=0.48, *P*=.63, and Cohen *d*=0.12; cognitive assessment: t_58_=0.87, *P*=.39, and Cohen *d*=0.23; course total: t_58_=0.19, *P*=.85, and Cohen *d*=0.05). However, it is worth noting that the YesVR group had higher mean OSCE scores across all scoring categories, although the differences were not statistically significant when compared to the NoVR group.

## Discussion

### Principal Findings

The integration of qualitative and quantitative findings provided a more comprehensive understanding of students’ experiences. While STAI, TAI, and perceived preparedness scores quantified student anxiety and preparedness levels, the interviews and focus groups offered insights into underlying factors. The theme of *knowledge of the exam environment* suggested that VR simulation may have familiarized students with the OSCE room, helping reduce uncertainty about the exam setting—one of the major themes linked to anxiety. While this study was not a randomized controlled trial, the mixed methods approach allowed for a deeper exploration of how students perceived the role of VR in their exam readiness, highlighting its potential as a preparatory tool.

The data showed that students who used the VR simulation had lower state anxiety scores compared to those who did not, but without a controlled study design, a causal relationship cannot be confirmed. Student interviews provided additional context that may help explain this observed difference. Intolerance to uncertainty is a significant risk factor for increasing anxiety symptoms [40]. However, *knowledge of the exam environment* was a recurring theme among the students, who mentioned that seeing and experiencing the exam room in advance was useful. Students reported rethinking their study strategies and adjusting their expectations accordingly, although it is unclear whether these changes were directly due to the VR experience. It could be that the small moment spent contemplating the exam setting outside of VR was enough to initiate their expectation adjustments. Regarding recurrent self-focused worry, *self-consciousness and insecurities* and *peer influences* were additional themes mentioned by students during the interviews, stating that their worries were caused by thoughts of inadequate performances while being watched. When practicing in real-world social situations, the students were aware of their potential mistakes and criticisms becoming known by peers. Student remarks such as “I didn’t want to fail because I didn’t want to look incompetent to my peers...” reflected this. While it was mentioned that having peer feedback provided assurances and important ideas for learning, some students said that they felt anxious when peer influences instilled a “we have to do this, this and this” mentality. Having the option to practice in a private VR simulation allowed students to make mistakes with no perceived consequences, potentially allowing them to hone their practice strategies and make mistakes without worry. The VR simulation may have offered a break from demanding peer objectives as they had a learning environment in which they could set the pace for themselves. Depending on how well they thought the VR simulation worked, students were persuaded by their peers to either participate or avoid the self-serve use of VR. The VR simulation was announced solely as a tool for potentially decreasing student anxiety—it was not advertised to raise students’ test scores. Some students may have perceived the VR system to be a less valuable source of learning after being informed that it was not designed to boost their grades. Examining student OSCE scores for this experiment revealed no differences between students who used VR and those who did not. The relationship between learning technology resources and student achievement is poorly understood and challenging to evaluate. The VR simulation would need to be significantly improved if the students’ only goal was to use it to raise their OSCE scores. Although there was a choice between 2 separate modules in the VR simulation, the system was not particularly thorough. Students could pose basic questions or try conducting a cognitive assessment on a virtual patient, but there was no feedback or scoring system. Such a system should be grounded in behavioral learning principles, include a wide array of patient scenarios, and provide scoring and reinforcement cues to help students understand how to improve their OSCE performance.

Regarding the handling of associated symptoms of anxiety leading to overanalyzing thought processes, the student themes of *feedback and support* and *time constraint* showed that students reported adjusting their questioning strategies while using the VR simulation, suggesting a possible link between VR sessions and changes in how students posed questions. The VR system glitched if students spoke with excessive dialogue. However, students noted that simplifying their questions to short, concise phrases seemed to boost their attentional focus. Some suggested that this helped them avoid overanalyzing and feeling anxious, although whether these outcomes resulted specifically from VR remains unclear. Greater anxiety levels are associated with overactivation of orienting processes, resulting in decreased capacity for attentional regulation [41]. However, the factors and directionality between manifested anxiety symptoms and attention are largely unknown [42]. Regarding the handling of rumination thought processes, themes of *framing* and *student grade mindset* emerged from the interviews and focus groups. When students adopt a positive and learnable growth mindset, there are perceptual changes in student aptitude, reducing anxiety about the course material [43]. The students in this investigation spoke of mentally changing the severity of the OSCE by saying either “the OSCE is only a learning experience” or “just pretend it’s a game,” which is different from learning growth mindsets. This shift in framing may have freed up students’ attentional resources, enabling them to focus more effectively on the immediate task rather than on future outcomes, potentially reducing rumination associated with test anxiety. However, the impact of a student’s *gaming mindset* on test anxiety cannot be conclusively determined from this study.

The TAI was not found to be different between the NoVR and YesVR groups. This suggests a similarity in general test anxiety traits between the groups. The TAI was created as a tool to assess test anxiety as a *situation-specific personality trait* according to the manual by Spielberger et al [33]. The study by Concannon et al [1] found this exact outcome between the NoVR and YesVR groups throughout multiple time points across the student curriculum. Consistent with trait test‑anxiety theory [5], students with higher trait test‑anxiety scores exhibit a stable, cross‑context anxiety response that surfaces in nearly all evaluative situations. In a report by Sargolzaei et al [44], a 21-minute, 360° instructional VR video for heart surgery students was able to reduce trait anxiety compared to a control group [44]. For the reduction of trait anxiety in internship settings, immersive VR interventions are recommended to be supplemented with instructional audio commentary and augmented reality notes [44]. For the reduction of trait anxiety in other anxiety-related disorders such as social anxiety, meta-analysis reports recommend using sophisticated VR exposure therapy across 4 to 14 sessions, each lasting 60 to 90 minutes [45].

Between 3 weeks and 3 days before the OSCE, all variables of perceived preparedness among YesVR students increased. A similar trend was reported in the study by Massey et al [11], which used video OSCE exemplars as blended learning materials for nursing students. They reported that a reduction in student stress can be attained by improving preparation and understanding of assessment expectations [11,46]. Of all the perceived preparedness variables, student-perceived confidence levels were found to be the lowest at both the beginning and the end of the OSCE preparation timeline despite showing a significant improvement (*P*=.01). Previous reports have stated that generation Y OT students (those born in the 1980s) exhibited overconfidence behaviors that reduced their openness to feedback from educators [47]. However, our investigation found student self-reported levels of confidence to be the lowest of all perceived preparedness variables, with *feedback and support* being a recurring theme mentioned by students to be important for their confidence development. This discrepancy could be due to a couple of factors. First, the students in our investigation reported that the large class sizes made it difficult to receive feedback. Increased class sizes and teaching workloads in OT means that new technology is recommended to be implemented in student curriculums with around-the-clock access [48-51]. This technology is recommended to provide feedback specific to each student, filling gaps in their knowledge based on performances in evaluative situations. Second, this investigation’s Perceived Preparedness Inventory measured OT student confidence of a specific type—perceived confidence in performance in an upcoming OSCE. The specificity of this confidence type is crucial. For example, a report by DeCleene Huber et al [52] found that increased clinical experience in OT students made an impact on how confident they felt when using evidence-based practice as future practitioners. Without this experience, occupational therapists would be less likely to perform evidence-based practice in clinical environments. However, the report by DeCleene Huber et al [52] also found that OT students showed less confidence when evidence-based practice skills were used in association with different tasks, such as statistical procedures and tests. Student confidence, knowledge, and skills have shown to be improved through motivational interviews and instruction [53]. Motivational interviews contain technical components (ie, open questioning, affirmation, reflective listening, and summarizing aspects) and relational components (ie, collaboration, evocation, autonomy support, and acceptance) [53]. One issue with these motivational interviewing sessions is that they typically have limited availability. However, immersive VR modules that offer simulated patient encounters could support interactive, experiential motivational interviews around the clock, which may enhance student perceived preparedness.

For OT student-perceived understanding, the *American Journal of Occupational Therapy* (2021) created a curricular design framework that recommended using formative assessment to measure student understanding while providing course adjustments and feedback throughout course progression [54]. These formative assessments can be in the form of simple, 1-minute reflections with *nonjudgmental* characteristics, allowing students to correct mistakes [55]. Activity resources reportedly improve student awareness levels, with technology playing a role in the form of web-based polls, student-uploaded pictures, videos, and comments on discussion forums [54]. The Perceived Preparedness Inventory was intended to probe respondents’ knowledge, understanding, awareness, skill level, and confidence surrounding OSCEs. The impact of our investigation’s immersive VR simulation was not specified in the Perceived Preparedness Inventory questions. Regardless of whether the students used the immersive VR simulation, it is possible that they could still experience higher perceived preparedness for their upcoming OSCE. The study by Concannon et al [1] found that student academic self-efficacy levels continuously increased throughout their first-year OT coursework regardless of whether the students had used VR simulation. Thus, it was beneficial to understand the degree to which each perceived preparedness parameter improved in a general sense.

### Future Recommendations

New cutting-edge components are available to enhance our investigation’s VR simulation and its general capabilities. However, adding new features based on student feedback from this investigation may enhance a student’s sense of immersion. The GPT AI can be upgraded to the latest version (ie, GPT-4o [omni] at the time of this writing) and trained on future recorded real-time interactions between student and patient actors. This would likely improve virtual patient response continuity for specific scenarios during the History Taking module. Regarding the reported lag time between student questions and virtual patient responses, using GPT-4o may reduce delays, although some models with simpler reasoning capabilities can generate replies more quickly. If used with a portable immersive VR headset such as the VIVE XR Elite, the simulation would be accessible around the clock in a portable manner with improved sound definition. Other technical improvements such as making the avatars more realistic (eg, rendered from Unreal Engine MetaHumans) while improving the animations of their movements may further support students’ sense of immersion. A greater variety of selectable avatars, including those from different cultures and backgrounds, might broaden student perspectives of real-world clientele while promoting diversity and inclusion.

Students were concerned about their training mistakes being known by their peers. It would be important for future VR learning environments to use disclaimers, informing users that their performances will not be recorded or used for assessment. From an ethical standpoint, if the VR simulation uses student interactions with virtual patients to train the AI system, this should be clearly informed to the student, and the ability to opt out should be offered. Overall, students said that their sense of preparedness for the OSCE was affected by their ability to access timely feedback and support during their clinical skill practice. This investigation found that, when students questioned the virtual patients during the History Taking module, it was beneficial to know when such questions became excessively worded. GPT is capable of providing condensed suggestions for such questions, which could be used to inform the student. Formative learning aspects of the simulation could also be improved to include AI assessment of interactions with the virtual patient, allowing the system to provide student feedback on the completeness of questioning procedures and evaluation of their bedside manner.

Programming virtual environments can be a costly and time-consuming process. However, if a module allowed the VR simulation to have its testing environments adjusted by the users themselves (ie, allow for positional adjustment of apparatuses such as desks and plinths), this would result in virtual test settings better representing those experienced in the real world. This would adhere to student preferences, who desire the practice environment to be similar to that of the actual OSCE.

### Strengths and Limitations

This investigation did not use a double-blind, randomized controlled trial; thus, causation of immersive VR’s ability to reduce student anxiety cannot be inferred. Conducting a randomized controlled trial could have been challenging as it may have disrupted the student study routines while potentially segregating them from their study partners. In addition, this investigation could not match student responses to demographic factors such as sex. However, this study was able to test students from the same academic year, potentially reducing group differences due to student admission requirements.

The overall response rate was relatively low. The respondents may not represent all enrolled students as those who chose to participate in the VR simulation might have been more open minded, performance focused, or generally eager to try new study methods, potentially limiting the broader applicability of these findings. Despite these limitations, the interview and focus group data offered meaningful context for the quantitative data, supplemented by student recommendations to understand their perceptions of immersive VR’s role in first-year health sciences coursework. Our qualitative aim was not to produce generalizable findings but, rather, to gain a deeper understanding and insight into student perspectives. We used focus groups and interviews to explore and generate themes; participants reiterated similar thematic categories, suggesting the data were approaching saturation; however, we do not claim full saturation, as additional participants or new cohorts might still reveal additional themes.

### Conclusions

Immersive VR simulation may help first-year OT students lower their state anxiety when preparing for clinical practical exams. Students reported feeling more oriented to the exam setting and found that VR sessions allowed them to practice questioning strategies in a low-stakes environment. One key theme was the need for additional practice on new exam components and student performance feedback. Future enhancements might incorporate automated scoring or simplified interview prompts for the students. Overall, VR could provide a safe environment for practice and feedback, potentially easing students’ anxiety in clinical test settings.

## Appendix

Multimedia Appendix 1 Interview guide.

Multimedia Appendix 2 Focus group guide.

Multimedia Appendix 3 Perceived Preparedness Inventory.

Multimedia Appendix 4 Saint Louis University Mental Status Exam.

Multimedia Appendix 5 State-Trait Anxiety Inventory raw data.

Multimedia Appendix 6 Test Anxiety Inventory raw data.

Multimedia Appendix 7 Perceived preparedness raw data.

## Abbreviations

AI: artificial intelligence
GPT: generative pretrained transformer
OSCE: objective structured clinical examination
OT: occupational therapy
SLUMS: Saint Louis University Mental Status
STAI: State-Trait Anxiety Inventory
TAI: Test Anxiety Inventory
VR: virtual reality

## Data Availability

The relevant quantitative datasets (State-Trait Anxiety Inventory, Test Anxiety Inventory, and perceived preparedness scores) generated or analyzed during this study are available in Multimedia Appendices 5, 6, and 7, respectively.

Although objective structured clinical examination score data were collected and assessed, releasing the full dataset could compromise student identity by making it possible to match specific scores to individuals. Therefore, these data are not publicly available but can be obtained from the corresponding author upon reasonable request.

The qualitative interview and focus group datasets are not publicly available because they contain researcher identifiers (eg, usernames and personal anecdotes) and fully deidentifying them for an open repository is unfeasible. However, key excerpts and themes have been included in the manuscript, and the underlying data are available from the corresponding author upon reasonable request.
