# Supplementary material for: Immersive Virtual Reality and AI (Generative Pretrained Transformer) to Enhance Student Preparedness for Objective Structured Clinical Examinations: Mixed Methods Study
Source: JMIR Serious Games. 2025 Apr 30;13:e69428. doi: 10.2196/69428 (PMC12079070; doi:10.2196/69428)
Supplement: Multimedia Appendix 2 [file games_v13i1e69428_app2.docx]

**Post OSCE Focus Group Guide**

Date: ________________________ Id # _________________________

1. Small Talk – rapport building

Introduction:

- Purpose of the Interview: to discuss with you your thoughts about the use of VR to deal with practical exams (OSCE)?
- Confidentiality reiterated
- Opportunity to end the interview at any time reiterated
- Any questions?

Thank participant for taking the time to participate.

The purpose of this meeting is to discuss your ideas, opinions and experiences on the **use of VR to help students manage anxiety associated with OSCEs.** What you discuss here today will be very helpful for our research project to understand student experience and the impact VR may have to help students cope with exam stress.

So that I do not miss any of your comments, I would like to record our discussion. I have asked for your permission to do this, as it will make my research work much easier. I should point out that your contributions will be anonymous and confidential, and that any published research will contain changed names.

Our discussion will last 60 mins max. During that time, I would like to explore a number of issues on this topic and hear everyone's responses. It would be better to keep your questions about this research project until the end, but please feel free to ask questions relating to the topic being discussed at any time. I’d like to start by asking you to introduce yourselves.

1. How would you rate your overall experience with the OSCE?
2. Was your experience in line with what you had expected?
3. What do you feel was the most difficult aspect of doing the OSCE?
4. How do you feel you managed your stress and anxiety while doing the OSCE?
5. What aspect of the OSCE do you find most stressful?
6. Now that you have completed the OSCE what strategies would suggest your course instructors use to help to make the exam situation less stressful or help student better deal with the anxiety of the exams?
7. Considering your experience with the VR simulation, did you find engaging in the activity helpful when preparing for your OSCE? Why or why not?
8. What aspects of the VR simulation did you find most helpful? What aspects did you find least helpful? (Can probe for exam preparation strategies, metacognitive strategies)
9. This VR simulation was developed to help students manage feelings of anxiety related to a new examination experience – the OSCE. In your opinion, did we accomplish this objective?
10. Do you have any suggestions for how the VR simulation can be improved?
11. How might you use this experience - preparing for the OSCE and participating in the VR simulation - for future OSCEs in the program?

**Wrap Up:** Any other comments? Any Questions? Thank you for your help.
