# Supplementary material for: Immersive Virtual Reality and AI (Generative Pretrained Transformer) to Enhance Student Preparedness for Objective Structured Clinical Examinations: Mixed Methods Study
Source: JMIR Serious Games. 2025 Apr 30;13:e69428. doi: 10.2196/69428 (PMC12079070; doi:10.2196/69428)
Supplement: Multimedia Appendix 3 [file games_v13i1e69428_app3.docx]

**Level of Preparedness for OSCE**

We appreciate your participation in our study. We are interested in obtaining some additional information that will assist with our study. Information gathered is anonymous and confidential.

**Note**: ‘1’ = very high, ‘2’ = high, ‘3’ = sound, ‘4’ = poor, and ‘5’ = very poor.

**Instructions:** Please select one of the following number-options for each question below:

Q1: My level of confidence for completing my Objective Structured Clinical Exam (OSCE) is:

1 2 3 4 5

Q2: My awareness of the expectations of the OSCE examiners is:

1 2 3 4 5

Q3: My knowledge of the content examined in the upcoming OSCE is:

1 2 3 4 5

Q4: My understanding of the OSCE examination process from the course material presented so far is:

1 2 3 4 5

Q5: My level of skill developed through the use of peer-review and feedback to others is:

1 2 3 4 5

**Thank you!**

This instrument was adapted from:

Massey, D., Byrne, J., Higgins, N., Weeks, B., Shuker, M. A., Coyne, E., ... & Johnston, A. N. B. (2017). Enhancing OSCE preparedness with video exemplars in undergraduate nursing students. A mixed method study. *Nurse Education Today*, *54*, 56-61.

Weeks, B. K., & Horan, S. A. (2013). A video-based learning activity is effective for preparing physiotherapy students for practical examinations. *Physiotherapy*, *99*(4), 292-297.
