# Supplementary material for: Immersive Virtual Reality and AI (Generative Pretrained Transformer) to Enhance Student Preparedness for Objective Structured Clinical Examinations: Mixed Methods Study
Source: JMIR Serious Games. 2025 Apr 30;13:e69428. doi: 10.2196/69428 (PMC12079070; doi:10.2196/69428)
Supplement: Multimedia Appendix 4 [file games_v13i1e69428_app4.pdf]

VAMC

SLUMS Examination

Questions about this assessment tool? E-mail aging@slu.edu.

Name ______________________ Age ______________________

Is patient alert? ______________________ Level of education ______________________

__/1 (1) **1. What day of the week is it?**

__/1 (1) **2. What is the year?**

__/1 (1) **3. What province are we in?**

4. Please remember these five objects. I will ask you what they are later.

Apple Pen Tie House Car

5. You have $100 and you go to the store and buy a dozen apples for $3 and a tricycle for $20.

(1) **How much did you spend?**

__/3 (2) **How much do you have left?**

6. Please name as many animals as you can in one minute.

__/3 (0) 0-4 animals (1) 5-9 animals (2) 10-14 animals (3) 15+ animals

__/5 **7. What were the five objects I asked you to remember? 1 point for each one correct.**

8. I am going to give you a series of numbers and I would like you to give them to me backwards. For example, if I say 42, you would say 24.

__/2 (0) 87 (1) 648 (1) 8537

9. This is a clock face. Please put in the hour markers and the time at ten minutes to eleven o'clock.

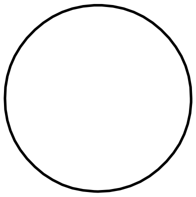

(2) Hour markers okay

__/4 (2) Time correct

(1) **10. Please place an X in the triangle.**

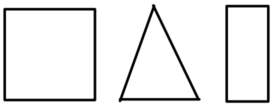

__/2 (1) **Which of the above figures is largest?**

11. I am going to tell you a story. Please listen carefully because afterwards, I'm going to ask you some questions about it.

Jill was a very successful stockbroker. She made a lot of money on the stock market. She then met Jack, a devastatingly handsome man. She married him and had three children. They lived in Toronto. She then stopped work and stayed at home to bring up her children. When they were teenagers, she went back to work. She and Jack lived happily ever after.

__/8 (2) **What was the female's name?** (2) **What work did she do?**

(2) **When did she go back to work?** (2) **What province did she live in?**

______ **TOTAL SCORE**

SCORING		
HIGH SCHOOL EDUCATION		LESS THAN HIGH SCHOOL EDUCATION
27-30	NORMAL	25-30
21-26	MILD NEUROCOGNITIVE DISORDER	20-24
1-20	DEMENTIA	1-19

CLINICIAN'S SIGNATURE ______________ DATE ______________ TIME ______________

SH Tariq, N Tumosa, JT Chibnall, HM Perry III, and JE Morley. The Saint Louis University Mental Status (SLUMS) Examination for detecting mild cognitive impairment and dementia is more sensitive than the Mini-Mental Status Examination (MMSE) - A pilot study. *Am J Geriatr Psych* 14:900-10, 2006.
